# Cutaneous squamous cell carcinomas secondary to hydroxyurea: a case report^؆^

**DOI:** 10.1016/j.abd.2026.501322

**Published:** 2026-03-27

**Authors:** Renata Guerra Galvão Santos, Marcio Martins Lobo Jardim, Lais Guerra Guedes, Isis Carla de Lima Pereira

**Affiliations:** Centro de Estudos Dermatológicos do Recife, Santa Casa de Misericórdia, Recife, PE, Brazil

Dear Editor,

A 73-year-old female patient with Polycythemia Vera (PV) who had been using Hydroxyurea (HU) for 16 years presented with multiple erythematous, keratotic plaques, papules, and nodules measuring up to 4 cm, some with central ulceration, located on the face and upper limbs for one year (Fig. 1A), followed by spontaneous regression of the lesions after HU discontinuation (Fig. 1B). Dermoscopy showed white circles, hemorrhagic spots, radial linear vessels, and punctate vessels (Fig. 2A).

**Fig. 1:**
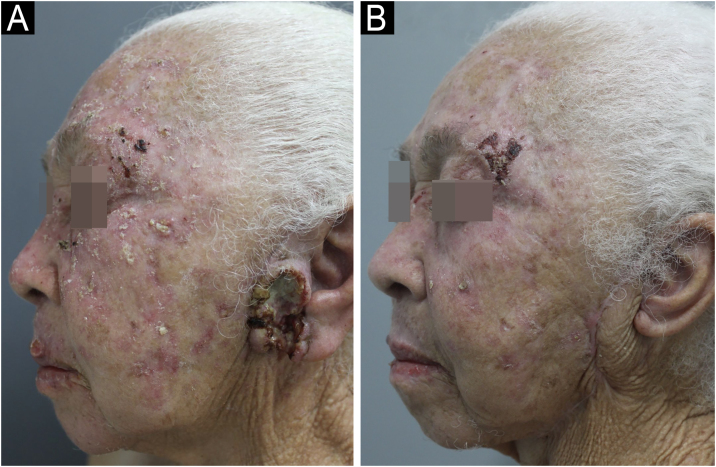
(A) Preauricular ulcerated tumor lesion and multiple hypertrophic actinic keratoses – during HU use; (B) Linear scar after surgical excision and significant regression of actinic keratoses on the face – six months after HU discontinuation.

**Fig. 2:**
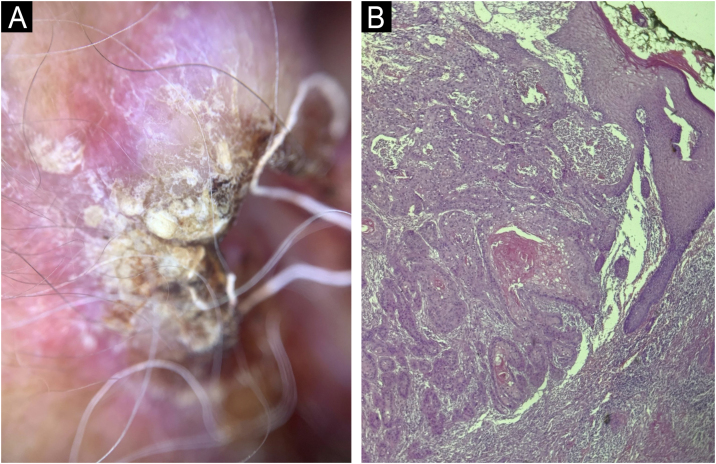
(A) Dermoscopy of the left preauricular lesion: white circles, radial linear vessels and punctate lesions. (B) Histopathology: infiltrative blocks of atypical keratinocytes in the dermis, corneal pearls, keratinization and dyskeratosis foci (Hematoxylin & eosin – ×100).

Excision was performed, with a 7-mm margin, on lesions located on the right nasal wall, in the left preauricular region, and three hyperkeratotic and ulcerated nodular lesions measuring up to 1.8 cm, located on the left upper limb, two on the forearm and one on the arm, of which anatomopathological (AP) studies showed well-differentiated Squamous Cell Carcinoma (SCC; Fig. 2B). As a course of action, it was decided to discontinue HU together with the hematology team. In the follow-up, after six and nine months, spontaneous and progressive regression of actinic keratoses and other tumor lesions on the face was observed (Figs. 1B, Fig. 3, Fig. 4), confirming the causal relationship between HU and the appearance of cutaneous carcinomas.

**Fig. 3:**
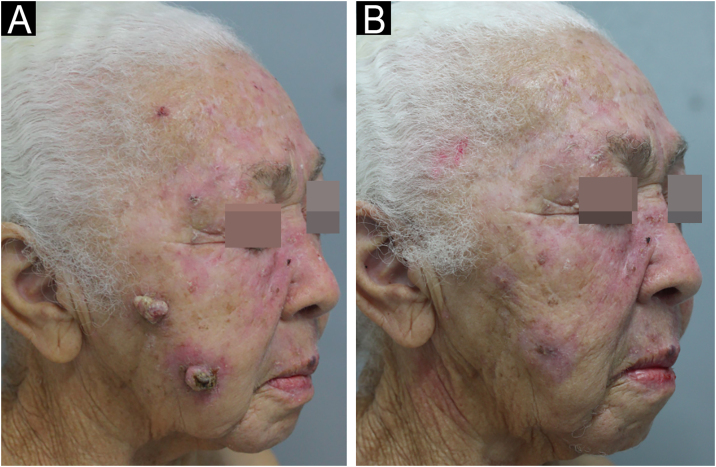
(A) Multiple erythematous, keratotic papules and plaques, and two ulcerated nodules – six months after HU discontinuation. (B) Spontaneous resolution of nodular lesions and improvement of actinic keratoses are observed – nine months after HU discontinuation.

**Fig. 4:**
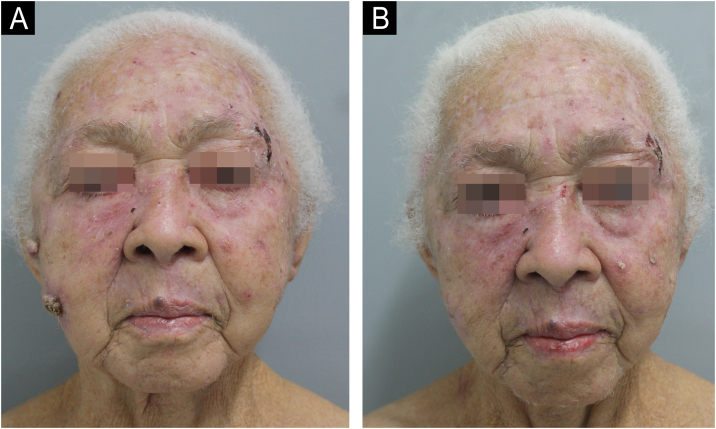
(A) Multiple erythematous, keratotic papules and plaques, and two ulcerated nodules – six months after HU discontinuation. (B) Spontaneous resolution of nodular lesions and improvement of actinic keratoses are observed – nine months after HU discontinuation.

HU is a cytotoxic drug used in the treatment of myeloproliferative diseases such as PV, essential thrombocytopenia, and primary myelofibrosis.^1^ The mechanism by which HU induces cutaneous carcinomas is complex and involves both the inhibition of DNA synthesis and repair in irradiated cells, leaving the strands exposed for longer and interfering with keratinocyte replication in the basal layer.

The medication decreases DNA polymerase activity, impairing the polymerization rate at DNA damage repair sites.^2^ In summary, HU apparently acts synergistically with ultraviolet radiation, promoting the development of aberrant p53 clones, causing non-melanoma skin cancers.^3^, ^4^ In literature reports, the multiple malignant skin lesions that developed acutely in these patients showed significant improvement after HU discontinuation.

In 2004, Sanchez-Palacios & Guitart^5^ reported two cases and reviewed 17 other case reports from the literature of patients with aggressive skin cancers associated with prolonged HU use. Among the studied patients, a sudden onset of cutaneous dysplasia was observed after a period of two to 13 years of drug use, with a predominance of male patients (10:7). In this review and in the reviewed studies, the patients’ age ranged from 59 to 83 years. Most patients showed improvement of the lesions after HU discontinuation. Only two cases did not show improvement, developing new cutaneous carcinomas two to four years after discontinuation.

In 2020, Bulte et al.^6^ also described that Non-Melanoma Skin Cancers (NMSC) often develop after a long period of HU use, with an average latency of 79 months, although lesions have been reported after only six months of use. Several types of pre-neoplastic and neoplastic lesions were observed after HU use, such as SCC, actinic keratoses, basal cell carcinoma, and Merkel cell carcinomas. The author also reported that patients experienced improvement or stabilization of lesions after drug withdrawal, and pointed out that in the case of NMSC development, the first therapeutic option should be HU discontinuation, followed by dose reduction or intermittent use.^6^

Finally, it is essential to closely monitor patients using HU, actively search for new skin lesions, and perform early biopsies of suspicious lesions. Follow-up should be maintained even after medication is discontinued, as multiple skin lesions may recur several years after drug withdrawal.^7^ Patients using HU should avoid excessive sun exposure, use photoprotection, and, if necessary, use chemopreventive agents such as oral retinoids.^1^

Stegelmann et al.^8^ emphasizes that the incidence of adverse skin events with HU use was twice as high in prospective studies compared to retrospective studies,^8^ highlighting the importance of educating patients about the possible side effects of the drug.

Despite the current data in the literature, robust multicenter prospective studies are required to clearly establish the causal relationship between HU and non-melanoma skin cancers.^1^

## ORCID ID

Renata Guerra Galvão Santos: 0000-0002-5201-5066

Marcio Martins Lobo Jardim: 0000-0002-8431-3607

Lais Guerra Guedes: 0009-0003-6776-1277

Isis Carla de Lima Pereira: 0009-0002-5345-2257

## Financial support

None declared.

## Authors' contributions

Renata Guerra Galvão Santos: Design and planning of the study; collection of data, or analysis and interpretation of data; drafting and editing of the manuscript or critical review of important intellectual content; collection, analysis, and interpretation of data; critical review of the literature; approval of the final version of the manuscript.

Marcio Martins Lobo Jardim: Design and planning of the study; effective participation in research orientation; intellectual participation in the propaedeutic and/or therapeutic conduct of the studied cases; critical review of the literature; approval of the final version of the manuscript.

Lais Guerra Guedes: Design and planning of the study; collection of data, or analysis and interpretation of data; drafting and editing of the manuscript or critical review of important intellectual content; collection, analysis, and interpretation of data; critical review of the literature.

Isis Carla de Lima Pereira: Collection of data, or analysis and interpretation of data; collection, analysis, and interpretation of data; critical review of the literature.

## Research data availability

Does not apply.

## Conflicts of interest

None declared.
